# MITE Digger, an efficient and accurate algorithm for genome wide discovery of miniature inverted repeat transposable elements

**DOI:** 10.1186/1471-2105-14-186

**Published:** 2013-06-07

**Authors:** Guojun Yang

**Affiliations:** 1 Department of Biology, University of Toronto Mississauga, Mississauga, ON, L5L 1C6, Canada

## Abstract

**Background:**

Miniature inverted repeat transposable elements (MITEs) are abundant non-autonomous elements, playing important roles in shaping gene and genome evolution. Their characteristic structural features are suitable for automated identification by computational approaches, however, *de novo* MITE discovery at genomic levels is still resource expensive. Efficient and accurate computational tools are desirable. Existing algorithms process every member of a MITE family, therefore a major portion of the computing task is redundant.

**Results:**

In this study, redundant computing steps were analyzed and a novel algorithm emphasizing on the reduction of such redundant computing was implemented in MITE Digger. It completed processing the whole rice genome sequence database in ~15 hours and produced 332 MITE candidates with low false positive (1.8%) and false negative (0.9%) rates. MITE Digger was also tested for genome wide MITE discovery with four other genomes.

**Conclusions:**

MITE Digger is efficient and accurate for genome wide retrieval of MITEs. Its user friendly interface further facilitates genome wide analyses of MITEs on a routine basis. The MITE Digger program is available at: http://labs.csb.utoronto.ca/yang/MITEDigger.

## Background

Miniature inverted repeat transposable elements (MITEs) are short non-autonomous transposable elements (TEs) that move by cut-and-paste mechanisms [1-3]. They do not produce transposases, proteins that mobilize TEs, and are therefore dependent on those produced by autonomous elements for transposition [4,5]. Compared to typical cut-and-paste transposons, MITE families often have high copy numbers, and transposition of these elements generates widespread genomic variations [6-8]. Due to their small sizes, MITE insertions are much less disruptive to genes than the larger elements. Therefore, they can often be found in genic regions, introducing phenotypical changes in some cases. For example, an insertion of a *Stowaway* MITE named *dTstu*1 in the flavonoid 3′,5′-hydroxylase (F3′5′H) gene of a potato leads to red pigmentation, and a *mPing* insertion in the rice *Hd*1 gene results in changes in flowering time [9-11]. Most MITE insertions may not cause phenotypical changes, but rather they alter gene expression levels and epigenetic profiles that may contribute to the overall fitness of the organisms under certain conditions [6,12-16]. While understanding how MITEs transpose to achieve high copy numbers can further our knowledge on their influence on genome evolution and provide MITE based genetic tools [4,17-19], genome wide identification and characterization of MITE families broaden our views on different types of MITEs and the scale of their activity and amplification during evolution [6,20-23]. New MITE families may become better candidates for studies of their transposition and amplification as well as for genetic markers.

MITEs were first discovered from the genetic variation caused by an insertion at the maize *wx*-B2 locus [24]. Computational approaches were employed to assist the characterization of MITEs with the increasingly available genomic sequences in databases around early 1990s [25]. Due to their well defined structural features including small size (50–800 bp), terminal inverted repeats (TIRs) and target site duplications (TSDs), the task to discover MITE families at genomic levels is suitable for automation. However, the complexity of higher eukaryotic genomes presents a major challenge for such automation. The TSDs and TIRs of MITEs are very short sequences that can occur at a high frequency by chance, resulting in a large number of false output entries that need to be manually analyzed [26-28]. Automated genome wide identification of MITEs can be time consuming because of the large sizes of higher eukaryotic genomes and high TE contents. Such computing tasks are often demanding on computing resources such as the number of CPUs and the amount of RAM.

The program FINDMITE was developed and used for the discovery of eight novel MITE families in the malaria mosquito *Anopheles gambiae* genome from a sequence database containing short entries [28]. Its input parameters include a predefined sequence or size of TSD, the length of TIRs, and the minimal distance between TIRs. All sequences satisfying the parameters are retrieved and processed. The program MUST, MITE Uncovering SysTem, is based on string matching to identify candidate TIR structures followed by checking the presence of a flanking TSD pair [29]. All candidates are retrieved and grouped. For genome wide analyses of higher eukaryotic MITEs, FINDMITE and MUST generate a large number of false positives because many sequences that satisfy the defined parameters can occur by chance. MITE-Hunter was developed to decrease the number of false positives [30]. It uses multiple sequence alignment to filter out sequences otherwise meeting MITE signature criteria but bearing similar flanking sequences. As a result, MITE-Hunter has a false positive rate of 4.4-8.3% compared to 85% of FINDMITE and 86% of MUST [30]. In these programs, all of the candidate elements were retrieved and analyzed while, theoretically, the identification of only a single element is necessary for a MITE family with hundreds of copies. Therefore the existing algorithms are resource expensive and require lengthy processing time. For example, it took MITE-Hunter ~44 hours to process the rice genome database (~380 Mb) with a Linux cluster using five CPUs. The 700 raw output entries were reduced and grouped into 132 MITE families with manual downstream analyses.

Here, an algorithm was developed to increase processing efficiency by reducing or avoiding redundant computing, therefore shortening the processing time and reducing the requirement for computing resources. The novel algorithm was implemented in MITE Digger, one of the few TE analysis programs featuring graphical user interface [31,32]. When tested with the rice genome sequence database, it took MITE Digger ~15 hours on a quad core Windows system to complete processing with a typical memory use of ~150 Mb. Comparative analyses of the MITE Digger output with the MITE Hunter output showed that MITE Digger is accurate with low false positive and false negative rates.

## Methods

### Database and programs

The rice genome sequence database was obtained from IRGSP/RAP build 5 [33]. The output from MITE-hunter was obtained from Yujun Han and Sue Wessler (personal communication) [30]. Analyses of MITE families and comparisons were performed with MAK1.8 [34,35] (http://labs.csb.utoronto.ca/yang/MAK/). MITE Digger was based on the Perl script used for the retrieval of ATon elements [36]. Blast + 2.2.24 was used to perform sequence similarity searches. MITE Digger output was generated using its default parameters. The MITE Digger entries that do not match those in the MITE-hunter output were searched against the rice repeat database using RepeatMasker (http://www.repeatmasker.org/cgi-bin/WEBRepeatMasker). Genome sequences of rapa, tomato, potato and sorghum were downloaded from plantGDB (http://www.plantgdb.org/).

### Implementation and testing system

The algorithm was implemented in ActivePerl 5.10.1 with Perl Tk 804.029 and Bioperl modules [37]. MITE Digger was tested on Windows XP and Windows 7 systems.

## Results and discussion

### Redundant computing in genome wide discovery of MITEs

A MITE family typically consists of several hundred highly similar copies. Therefore, when every candidate element is processed in genome wide analyses, a MITE family can be computed hundreds of times. Such repetitions can occur at multiple stages including signature feature (i.e. TIR and TSD) identification, screening, multiple sequence alignment or clustering. These repetitions also occur to the elements that do not qualify for the input criteria such as the element length. Furthermore, genomes often contain highly repetitive sequences such as retro elements that have the structures of short inverted repeats flanked by short direct repeats buried in their internal sequences. These non-MITE sequences can occur more often than MITE families and each family may be computed hundreds or thousands of times depending on their copy numbers.

These redundant computing can take up a major portion of the total processing time. In addition, after retrieval of every element of a family, the need to remove redundancy or to group elements into a family can be time consuming and resource intensive. Particularly, when multiple sequence alignment is used to identify candidates with different flanking sequences, aligning elements in a family with several hundred copies takes a significant amount of time. Finally, when database entries are sliced into very short fragments to reduce memory use, the processing efficiency can be dramatically affected because of the overhead on retrieving and analyzing a large number of sequences.

### Measures to reduce redundant computing

**Figure 1:**
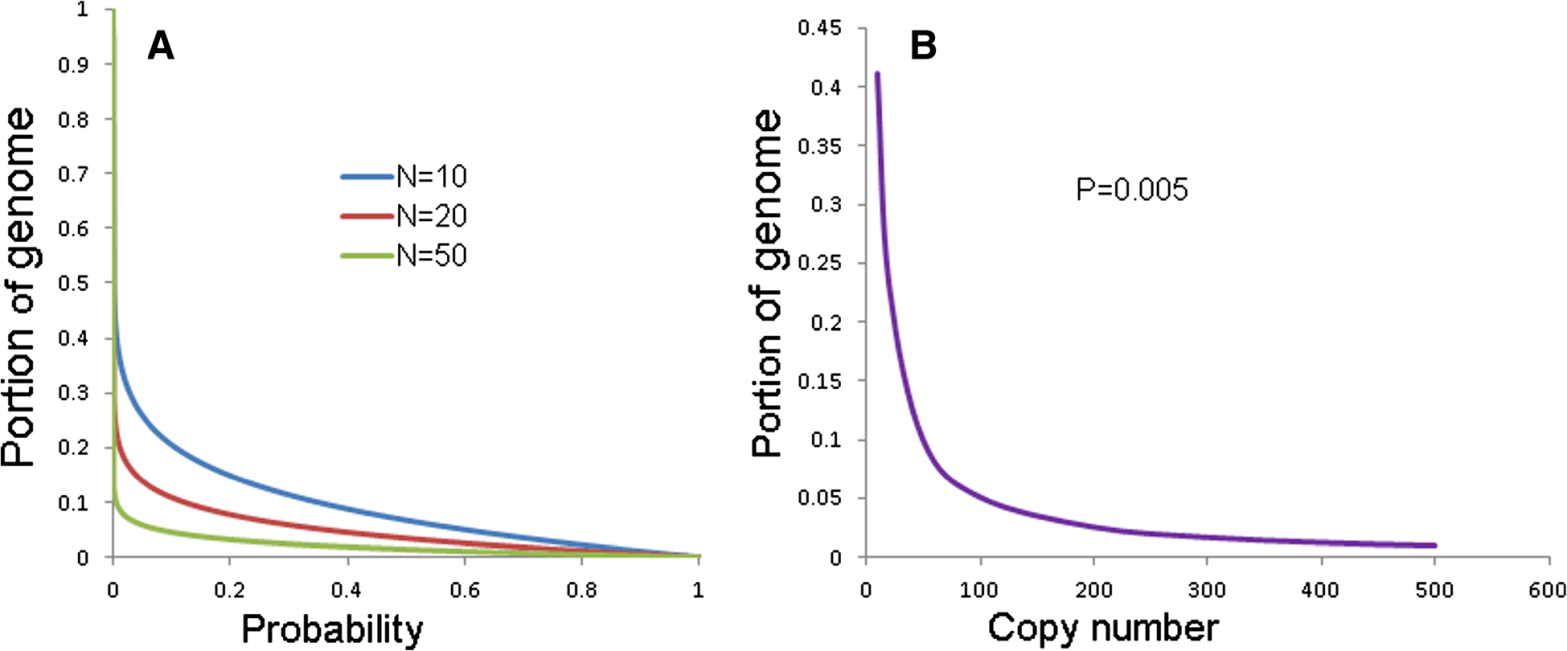
**Analysis of redundant computing in genome wide MITE discovery.** (**A**) The portions of a genome sequence database at which to miss an element of a MITE with N (10, 20, 50) copies at different probability levels; (**B**) The portion of a genome sequence database at which to miss an element with a probability of P = 0.005, i.e. P = 0.995 to contain an element, for families with different copy numbers.

Elimination or reduction of these redundant computing should dramatically increase efficiency. Theoretically, only a single complete copy of a MITE family is sufficient to represent the whole family and repetitions on other copies are unnecessary. Therefore, computing on each member of a candidate MITE family or a false family can be reduced to the computing on a representative of the family. Once a representative is identified, later occurrences of the family can be filtered out from the database entries. The redundancy is heavily concentrated toward the later part of a genome sequence database. If the members of a family with N copies are distributed randomly in a genome, the probability (P) of not having any copy present in the first X portion of the genome is: P = (1-X)^N^, therefore the portion of the genome to have at least one copy at this probability can be calculate as X = 1-P^1/N^. The portion of a genome to have at least one copy of a family with 10, 20, 50 copies at different probability level can be calculated (Figure 1A). Accordingly, the probability of missing an element of a MITE family with 10, 20, or 50 copies in 37%, 21% and 8% of a genome database is only 0.01, i.e. the probability to contain at least one copy of these families in respective portions of the genome database is high (P = 0.99). Less than 5% of the genome database is needed to contain at least one copy for MITE families with >100 copies with the probability of 0.995, i.e. probability of 0.005 to miss (Figure 1B). Therefore, only a small portion of a genome may be necessary to retrieve most of the MITE families; this is particularly useful in the processing of large genome databases. The members of a MITE family are normally expected to be located in different genomic loci, a false family can be identified as that having similar flanking sequences for every individual copy. Instead of an alignment of the flanking sequences of all copies of a family, comparison of those of a small subset of the members is sufficient and can reduce processing time dramatically. In addition, the removal of low complexity regions at start can eliminate the need to process false TIR and TSD motifs that are often found in such regions. Finally, processing the database entries in large sizes with high sensitivities will reduce the overhead of processing numerous small entries.

### Pipeline algorithm and parameters

**Figure 2:**
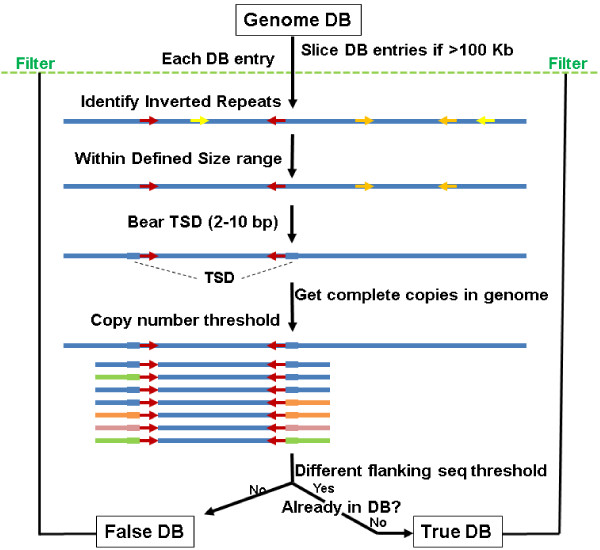
**Algorithm of MITE digger.** Arrowheads of the same color, inverted repeat pairs; Colored lines flanking inverted repeat pairs, different flanking sequences. DB, database; TSD, target site duplication.

MITE Digger takes a genome sequence database file in FASTA format as input. The entries are automatically sliced to the maximal size of 100,000 nt if the entries are larger than this size (Figure 2). Each entry of the sliced database was used to search against a reverse complementary sequence of the entry to reduce memory consumption and increase sensitivity. This search was performed with highly sensitive parameters (e.g. evalue 10000) to reveal very short inverted repeats. Tabular output format was used to reduce memory use. Simple inverted repeats such as those with stretches of a single or dinucleotides and those only contain G/C or A/T were ignored. A pair of short stretches of sequences (10 nt) flanking the inverted repeats within the defined length range (e.g. 50–800 nt) were used to find direct repeats of 2 to 10 nt in size. Since TSDs of some elements such as *Stowaway*s and *PiggyBac* TEs may be present in inverted repeats, the search for direct repeats is also performed on the most terminal 5 nt of the inverted repeats if direct repeats are not found in the flanking sequences. The DNA sequences between each qualified inverted repeats were then used to search against the whole genome database and the number of full length copies were counted. When a candidate meets the copy number threshold, the flanking sequences of 50 nt on each side were retrieved. The flanking sequences of different copies of the candidate family were compared. When qualified for the number of different flanking sequences, the candidate sequence was deposited into the filter database and the output if a similar sequence is not already in the output. Otherwise, the sequences meeting the copy number threshold but not the number of different flanking sequences are deposited into the filter database and the false output. The filter database is used to mask the upcoming genome database entries. The processing information is printed to a report file each time a new candidate is identified. When a new candidate or false sequence is deposited, the filter database and the output database (as applicable) were reformatted for BLAST searches.

### Performance analyses

**Figure 3:**
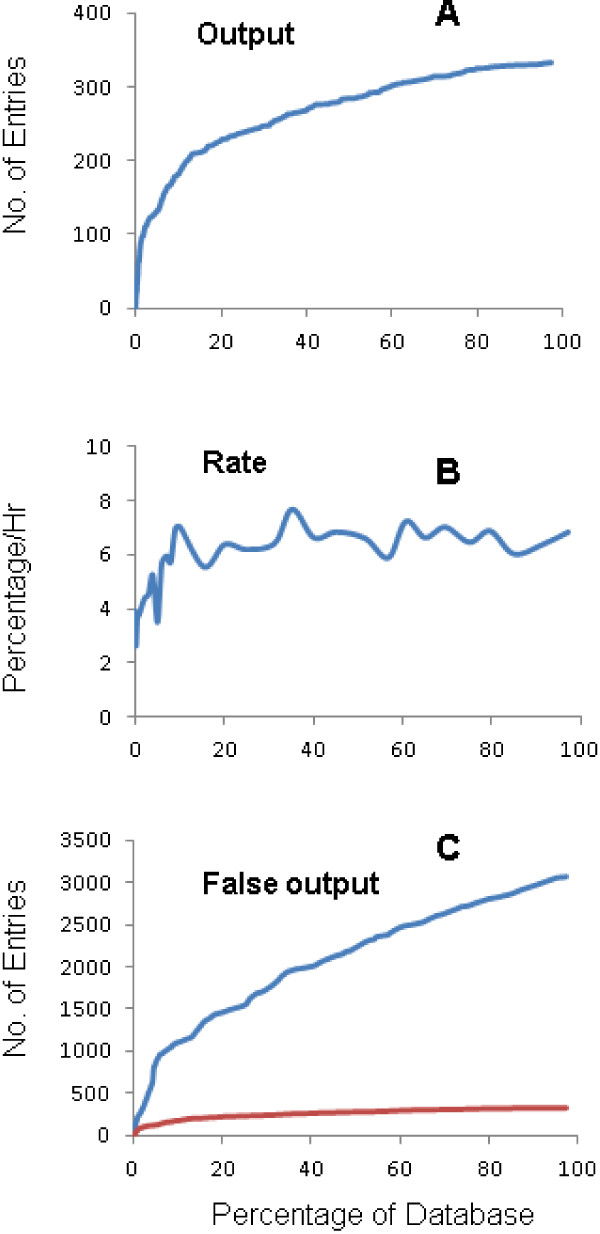
**Processing of the rice genome by MITE digger.** (**A**) The number of entries in the output database at certain processed portion of the database; (**B**) The processing rate, expressed as the percentage of the database processed per hour, at certain database percentage; (**C**) The number of entries in the false output database at certain processed portion of the database. Red, the number of entries in the output shown in (**A**).

MITE Digger was used to process the rice genome database to identify MITEs with a size range of 50 to 800 nt. On a Windows XP system with 4 CPU cores, processing finished in 15.44 hours with a typical memory use of ~150 Mb. A total of 332 candidate MITE sequences and 3071 false sequences were generated (Additional file 1). Entries from MITE Digger output can be used directly for downstream analyses. When the numbers of output entries were plotted against the portions of the genome processed, it is apparent that the number of candidates retrieved in a certain amount of genome sequences decreases with the increase of the amount of processed genome sequences. This reflects the reduction in redundant computing (Figure 3A). The most apparent increase in the number of candidates is in the first 20% of the genome. The curve nearly flattens out for the last 20% of the genome. While the processing rate fluctuates because of different levels of complexity of sequences in different regions, it clearly more than doubled from <3% of the genome sequence database per hour at the beginning to >6% at the end of the processing (Figure 3B). Predicted remaining time to completion decreases dramatically particularly during the first 10% of the genome database. Filtering has also dramatically reduced the number of false elements per database unit over the course (Figure 3C).

MITE Digger allows customized input of parameters. Database entries larger than the defined entry size will be automatically sliced and automatically formatted. The option to set the number of CPUs allows optimal performance of MITE Digger in platforms with different hardware settings. The option for probability level allows timely processing of large genome databases with a minor chance of missing a MITE family. Changes to other parameters such as the copy number threshold, different flank sequence threshold and sensitivity will affect the number of output entries. The predicted running time is based on the current average processing rate, therefore the actual total run time can be dramatically shorter than the predicted time at the early part of processing because of acceleration (Figure 3B).

### Evaluation of MITE Digger output

The output from MITE Digger was compared with that of the MITE Hunter. First, the entries in the MITE Digger output were cross matched with those in the MITE Hunter output, resulting in 1407 non-redundant matching pair records. Since the MITE Hunter output contains entries up to 1500 bp, only those between 50 and 800 bp were considered as MITEs. Among the 1407 records, 658 pairs cover at least 80% of the length of both query and hit sequences. The remaining records were manually inspected and four additional matching pairs were found. Because a MITE family may contain several subfamilies, one entry from MITE Digger can match several entries in MITE Hunter and *vice versa*. Therefore, the 662 matching pairs consisted of only 287 MITE Digger entries and 301 MITE Hunter entries (Additional file 2). In the remaining 749 records, 13 MITE Digger entries match the terminal sequences of some MITE Hunter entries, suggesting that these MITE Digger entries represent new subfamilies in the families of the MITE Hunter entries (Additional file 2). The rest of the records match short regions in the internal sequences of some long entries of the MITE Hunter output and were considered non-informative records. Therefore, of the 332 MITE Digger output entries, 300 can be classified with the MITE Hunter output entries. The remaining 32 entries were scanned with rice repeat database using RepeatMasker, 11 of them were previously annotated elements or can be manually classified. Six (1.8%) of them are false positives: two rice simple repeats and four retroelements (one SZ-50_int-int LTR terminal, two Cassandra, and one SINE03_OS). The other five were DNA elements: two *Stowaway*s (TREP215, STOWAWAY10_OS); one *Harbinger* (ID-4), one *Mutator* and one unknown category (OSTE23). Therefore a total of 17 (7.5%) MITE Digger output entries are classifiable MITE families that are not in the MITE Hunter output (Additional file 2). The remaining 21 entries cannot be classified even though they have the characteristics of Class II TEs.

To estimate the accuracy of MITE Digger, the output was compared with the MITE entries in Repbase and the entries in MITE Hunter output. Repbase contains 165 entries of non-autonomous Class II elements shorter than 800 bp. Among these sequences, only 19 did not have a match in the MITE Digger output. None of these elements meet the criteria for MITE: five (CASMALL, F804, HELIA, POP-OL2, WUJI) do not bear TIRs and the other 14 (CASIN, ECSR, GLUTEL1LIKE, HEARTBLEEDING, ID-2, NONAME, OSTE26, STONE, TOURIST-XIII, CACTA-G1, LIER, TAMI2, THRIA, TYPEU3) have less than 10 complete copies in the genome. Therefore, all of the MITE entries in Repbase were retrieved by MITE Digger. The accuracy was also calculated as the percentage of the MITE Digger output entries matching entries of the MITE Hunter output in the same size range (i.e. <=800 bp and > =10 copies in this case). Of the MITE Hunter entries, 462 are shorter than 800 bp and 239 of them also have at least 10 full length copies. Of these 239 MITE Hunter entries, 221 entries were found in the MITE Digger output, resulting in a missing rate for MITE Digger of 7.5% (18/239) (Table 1). Close inspection of these 18 elements revealed that, with the exception of OS_mhAT99 and OS_mhAT23, all of them have a significant part (50 – 242 bp, or 9%-87%) of their sequences matching an entry in the MITE Digger output (Table 1), suggesting that these elements were missed because elements of a different subfamily are present in the MITE Digger output. The reason OS_mhAT99 and OS_mhAT23 were missed remains unknown.

**Table 1:** Summary of the comparison between the MITE digger and MITE hunter output

**Category**	**MITE hunter output**	**MITE digger output**	**Matching entries**	**Only in hunter**	**Only in digger**
<= 800 bp	462	332	(Digger-Hunter)		
MITE (<=800 bp, >10 copies)	239/462	332	287-301		
CACTA	2/3	1	1-2		
mhAT	47/102	49	48-60	5	1
mMariner/Stowaway	64/95	91	88-70	6	3
mMutator	58/134	78	70-76	7	8
mPIFHarbinger	68/128	85	80-93		5
<10 copies	223				
>800 bp	75				
False positive (retro, simple)	58/700 (8.3%)	6/332 (1.8%)			
False negative		3/332 (0.9%)			
Unclassified	15/700	22/332 (6.6%)			
Compound/Fusion	26/700				
Redundant	50/700				
Missing%				=18/239 (7.5%)	=17/226 (7.5%)
Total	700	332			

To calculate the false negative rate, the entries of the MITE Digger false output were cross matched with the MITE Hunter output entries. A large number of matching pairs are those matching the internal regions of MITEs of the MITE Hunter output as expected. Only three MITE Digger false output entries were found to be real MITEs that matched MITE Hunter output entries (OS_mMutator_126, OS_mMutator_69, OS_mMutator_67), therefore the false negative rate is 0.9% (3/332).

To see how fast MITE Digger runs with other genomes, the recently released genome databases of banana (*Musa acuminate*), potato (*Solanum tuberosome*), tomato (*Solanum lycopersicum*), Chinese cabbage (*Brassica rapa*) and sorghum (*Sorghum bicolor*) were used. Genome wide characterization of MITE families has not been previously performed in these genomes. All of these genomes gave much fewer numbers of entries than rice in the MITE output (Table 2). It is apparent that the running times for the databases are not simply determined by the size of a database. The factors affecting running time include the size and complexity of a genome, the number of entries in a database, the number of candidate and false entries.

**Table 2:** MITE digger processing of additional genome databases

**Genome**	**Genome DB size (Mb)**	**Processing time**	**No. output entries**	**No. DB entries**	**No. false output entries**
*Brassica rapa*	268	19.76	85	42919	4111
*Solanum tuberosome*	567	37.06	134	5832	6997
*Solanum lycopersicum*	716	57.97	113	7425	9134
*Sorghum bicolor*	652	21.35	227	6267	3946
*Oryza sativa*	388	15.44	332	3734	3071
*Musa acuminata*	383	12.57	28	3930	1992

## Conclusions

In summary, MITE Digger retrieved exemplars of MITE families from the rice genome with high accuracy and low false positive and false negative rates. Importantly, MITE Digger is not computing resource intensive and the output requires minimal manual processing. Therefore, it can be used routinely to perform genome wide identification of MITEs in higher eukaryotic genomes.

## Availability and requirements

Project Name: MITE Digger

Project homepage: http://labs.csb.utoronto.ca/yang/MITEDigger

Operating system: Windows

Programming language: PERL

Other requirements: N/A

License: by the developer

Any restrictions to use by non-academics: license needed

## Abbreviations

TE: Transposable elements; MITE: Miniature inverted repeat transposable element; TIR: Terminal inverted repeat; TSD: Target site duplication.

## Competing interests

The author declares that they have no competing interests.

## Authors’ contributions

GY developed and implemented the algorithm, performed tests and analyses, wrote the paper.

## Supplementary Material

Additional file 1 MITE Digger report and output for the rice genome database. Click here for file

Additional file 2 Comparison of the output of MITE Digger and MITE Hunter. Click here for file
